# A Clinical Lecture on the Use of Calomel in Remittent Fever

**Published:** 1849-12

**Authors:** 


*A Clinical Lecture on the Use of Calomel in Remittent Fever.*—By W. G. Ramsay, M. D. (*Concluded from last number*).*

Another popular reason for the indiscriminate use of calomel, remains yet to be considered. After the bilious discharges which take place at the commencement of fever, have abated, it is said that the liver is in a state of torpor, and must be aroused to action—by mercury. To show the great absurdity of such an opinion, it is only necessary to consider the cause, why the liver does not secrete. It is an axiom in pathology, which is proved by facts of every day's occurrence, and which has never been doubted by any except those who discard pathology, as useless, "that the effect of inflammation on the secretions of the glands depends on the intensity it assumes; when it is slight, the secretion is augmented, and differs little, if any, from its physiological state. With a slight increase of inflammatory action, it is less perfectly elaborated, when the inflammation passes to a higher degree, the secretion diminishes and becomes highly acid, and is often mixed with blood. The inflammatory action acquiring a more active state, the secretion is entirely suppressed in the parts in which the inflammation exists. This being the fact, and as we know that all glands are governed by the same laws, can you reasonably expect to provoke the liver to secrete by stimulating it to action, when its secretions are arrested by a high grade of inflammation. This position is a strong one, and difficult, if not impossible to be got over, by those whose reason for administering mercury in fever, is to promote the secretions of the liver.

It is a well known fact to all of you, that the first effect of catarrh is to increase the discharge from the schneiderian membrane, and when the catarrhal symptoms are violent, the secretions are arrested; the persons suffering from headache, dryness of the nares and throat, and the common expression is, that the cold is in the *head.* Can you relieve these unpleasant symptoms by the application of irritating errhines? I answer, no. You would not for one moment think of such a remedy, if you understand the cause of these symptoms. What then, I would ask, constitutes the difference between the nature and indications of cure, of the familiar case I have just cited, and the diminution and often total suppression of the secretion of bile, during the high grade of bilious fever. The only differ-

---

* See page 349.

ence is this. That in the first case, if you irritate farther the schneiderian membrane, you will see the error of your practice; but in the latter, the injurious effect of the medication is too often put down to the violence of the disease. What are you to do to promote the secretion not only of the liver, as is erroneously thought to be the sole and only gland affected, but of the whole glandular apparatus. Reason and judgment will dictate the answer to every reflecting mind. You are to endeavor to remove the cause—which is inflammatory action.

To show you how erroneous it is to believe that a person dies of fever from a derangement of the liver and its secretions, I need but refer you to the many cases of "jaundice" we daily witness, where the bile is visible in nearly every part of the body, and yet the patient suffers few, if any unpleasant symptoms. Dr. Stokes, physician of the Meath Hospital in Dublin, relates two cases of jaundice, one lasting 18 months, and the other 16 months, Dr. Blundell relates two cases of children, who lived for 4 months apparently well-fed and healthy, when, on opening their bodies it was found that the biliary ducts terminated in a cul-de-sac—that is, a blind pouch. Sir Everard Home gives a remarkable case of the total absence of the gall bladder occurring in connection with a perfect state of health.

Here, then, are real facts which go far to show how erroneous it is to direct the treatment of fever to the correcting and restoring of the secretions of the liver, as the great cause of the fatality of fevers, and to rely on calomel as a specific to effect those purposes. The purpose for which mercury is given by the majority of practitioners, is to produce salivation, or the action of the remedy on the glandular system. Let us now examine and see if the means employed to effect this purpose are in accordance with the pathological state of the system when laboring under an attack of fever.

The advocates of this practice commence their treatment with calomel, and continue its exhibition until either the patient dies, the disease yields, or until the mercurial irritation is well developed, showing itself by the salivation which ensues. Enormous quantities of calomel have been administered before this purpose is effected. There are instances where 2000 grains of calomel have been taken without producing salivation. How does this happen? The advocates of this doctrine do not explain the reason, but if we are conversant with the laws of physiology, the explanation of this phenomena is not difficult to be understood. When salivation is produced, the modus operandi of the medicine is by "absorption." Now it is a well established fact, that the activity of absorption appears to be in relation to the state of the circulation. When the vessels are plethoric, absorption languishes, and it acquires great activity by the diminution of their contents. This fact is clearly established by the experiments of "Magendie," who found an artificial plethora produced by injecting water into the veins, to prevent the poisoning of "nux vomica" inserted under the skin, and which ensued in shorter periods, according to the quantity of blood abstracted by bleeding. This, then, further explains the reason why calomel given in fevers will not affect the salivary glands until the inflammatory symptoms have been reduced by depletion. The salivation is more an effect than a cause of the abatement of fever. This fact has been too little appreciated by the majority of physicians, but of late we have had some valuable information on this subject, which will tend in a great measure to rectify this practice in fevers. Dr. Stokes, in a clinical lecture at Meath Hospital, remarks:—

"Many persons are of opinion that it is the ptyalism which carries off the disease, and hence it is, that we so often see the principal share of a practitioner's attention directed to produce salivation at all hazards. Cases are on record, of 1000 or 2000 grains being given without producing salivation. You give one dose, which is soon followed by another, without producing ptyalism. All this is wrong practice." "Salivation," continues he, "is to be looked upon more as the result of the relief of inflammation, than as its cause. The greater the intensity of the disease, the less the chance of salivating. Intense inflammation has a great tendency to prevent the action of calomel on the salivary glands."

Dr. Rush, who believed that salivation was beneficial in fevers, from its evacuant effect, substantiates the point which I have attempted to prove. He remarks, in his Medical Observations and Inquiries,

"Mercury seldom salivated until the fever intermitted or declined. I saw," says he, "several cases in which the salivation came on during the intermissions, and went off during the exacerbations, and many in which there was no salivation until the morbid action had ceased altogether in the blood vessels, by the solution of the fever."

Dr. Daniel, in his work on the autumnal fevers of Savannah, makes the following observation on the above passage:—

"It is remarked by Dr. Rush," says he, (and who has not witnessed the same?) "that salivation which exists during the intermissions of a fever, is sometimes wholly suppressed by the paroxysm. If fever has the power of suppressing mercurial action, it certainly has the power to prevent it. I have witnessed, continues he, a few cases where a salivation supervening in the early stage of fever has, without in any way mitigating the disease or shortening its duration, run on with it for many days, much to the distress of the patient and annoyance to the physician. I have further known fever to supervene in persons laboring under salivation, produced in the treatment of chronic diseases, where ptyalism had subsided upon the supervention of fever, and returned upon its termination, without the intermediate administration of a single grain of mercury—as the following case will show. "A man who had passed a considerable portion of the preceding eight months in the Hospital, with an extensive ulcer in the groin, was admitted in the fall of 1822, with the same affection. It had been treated as syphylitic, and he had been salivated several times. A wash of lime water and calomel was directed by me, which in a short time produced a profuse salivation. In a few days after, he was attacked with remittent fever; upon the supervention of which, all symptoms of salivation wholly disappeared. The fever terminated in nine or ten days, upon which the salivation returned with its full force, with mercurial fætor, swelled salivary glands, and tongue, and inflamed gums, &c. Not one grain of mercury was administered to the patient after the supervention of fever."

This case, then, is one of great interest, and seems to show that fever will often suppress mercurial action already existing. I witnessed a case illustrative of this fact, in this institution, during the last winter. A patient was admitted with intermittent fever, of a quotidian type. The paroxysms were very severe, and depletion was carried to some extent; the paroxysms continued to occur every day, with less severity. During this time, he took a small portion of calomel combined with opium, when a profuse salivation took place. The paroxysms continued as usual, without any interruption, for some time after the salivation, and did not abate until he was put under the exhibition of quinine. This case was one of great value to me; and since then I have met with many others reported by different authors. In the American Journal, volume 5th, containing Jackson's clinical reports of cases in the Philadelphia Alms House, I find the following pertinent remarks on this subject, which will not fail to be highly useful to you:—

"The unfortunate result, says he, of attempting to cure fevers by the establishment of salivation, or to place the economy under the comprehensive denomination of the mercurial irritation, is, I have strong grounds to believe, a circumstance far more common than suspected, or than many will be disposed to acknowledge. This fashionable practice I have abandoned since the epidemic of 1822—the commencement of a series of epidemic intermittent and remittent fevers, that continue still to prevail over so large a portion of the country. I was compelled to renounce it, from witnessing, in so many instances, the injurious results of the treatment to the patient. The mercurial action, I found, in violent cases, could very rarely be brought on before the intensity of the local inflammation and the sympathetic fever were on the decline, and then the inflammations caused by the mercurial action were not to be desired; they were nearly as much to be dreaded as those which constituted the disease. In many cases, after convalescence had commenced, the mercurial action came on, and I had the mortification to be perfectly convinced—though no suspicion crossed the mind of the patient—that a rapid recovery had been prevented, and protracted suffering been endured, in consequence of the employment of the remedy, though done under the sanction of high authority at home, and great NAMES abroad. The mercurial irritation is to be kept in mind, when it is developed as febrile disturbances in the system are subsiding. It does not, in numerous instances, induce salivation, nor is this effect a necessary consequence of the administration of mercurial preparations; on the contrary, they often excite at that time, from the numerous irritations still existing, an extent of action in the mucous tissue of the digestive canal and the mouth, and which is thence extended into the glands connected with them. The consequence is inflammation, ulceration, hemorrhage—together with excitement of febrile commotion. The new train of symptoms are then frequently drawn on to a relapse, and, if the mercurial treatment be instituted, the patient almost assuredly perishes."

This shows you that the mercurial irritation is going on to the aggravation of the disease, without showing itself by salivation. In the treatment of fever there are a few important questions which every person ought to ask himself. Whether bilious fevers cannot be cured without submitting the patient to the liability of being salivated, with so much pain and uneasiness? Is calomel a specific in fever, without which it is vain and useless to attempt a cure?—*Charleston Med. Journal.*

